# Metallotherapy

**Published:** 1882-01

**Authors:** 


### METALLOTHERAPY.

M. Vigouroux, the assistant of Charcot at La Salpetriere, in a paper of considerable interest, collects what has been discovered, and at various times communicated to the profession, with reference to *metalloscopy*, *metallotherapeutics*, *and æsthesiogenics in general.* In 1876 M. Burq reported to the Societe de Biologie the fact that the application of a metal (generally in the form of a disc, like a piece of money) to the skin of an anæsthetic patient brought about a return of sensibility, which, however, was in most cases only temporary, the anæsthesia recurring after a variable interval, or after the removal of the metal. Different metals were efficient in various degrees ; and this was also found to vary in different individuals, some being susceptible to one metal, others to another. The name *metalloscopy* was applied to the subject, including also the various effects upon circulalation, temperature, etc., following the application of metallic plates to the surface. By *metallotherapeutics* is meant the therapeutic application of metalloscopy. At this stage of the matter M. Burq conceived the notion that metals applied to the mucous surfaces—that is, given by the mouth—might have a similar influence ; for instance, in a case where gold applied to the surface temporarily relieved anæsthesia, gold leaf, rolled into pills and administered by the mouth, might also relieve the anæsthesia. This he called *internal metalloscopy* ; and with true French enthusiasm he depicted the grand results which might be anticipated from combined external and internal metallotherapeutics, the disease being, as it were, brought between two fires. Further experiments revealed the fact that other substances beside metals possessed the power of restoring sensibility in anæsthetic parts, such as guttapercha, wood, collodion film, etc. ; and also different forms of electricity were observed to produce the same results. A wider term than *metalloscopy* being therefore required to include these new agents, *æsthesiogenics* (æsthesiogenes) was decided upon as a generic term, which should include and be applicable to all possible agents or influences which could restore sensation in anæsthetic parts. It is evident that the term *æesthesiogenics* refers to the agents rather than to the process itself, and also that it contains no allusion to effects upon temperature, etc. ; but the results of investigations have been so various and surprising that it is hard to conceive of a single word which would embrace and classify them. M. Burq discovered that the application of certain metals restored sensation in anæsthetic parts ; he also observed that in anæsthetic patients who wore thimbles, rings, bracelets, etc., the skin was often sensitive in their vicinity. The metal which is suited to a given case must be ascertained experimentally, a number being tried until an efficient one is hit upon. If a metal which at first restores sensation be kept for a long while in contact with the skin, anæsthesia in most cases finally returns (*anæsthesie de retour*). While M. Burq's investigations had reference exclusively to general sensibility, Charcot went further. Having already called attention to the fact that in cases of hysterical hemianæsthesia there were generally also present on the same side amblyopia, loss of taste, diminished muscular power, diminished temperature, color-blindness, diminished hearing power, etc., he now showed that the application of a suitable metal caused these various troubles to disappear simultaneously with the anæsthesia with which they were associated. It may be observed here that Charcot's experiments are all made upon hysterical patients. Charcot further demonstrated the *transfer* (*transferts*) and *oscillations.* In a hemianæsthetic patient, while the application of a metal was causing her various troubles to disappear from the side originally affected, it was observed that they reproduced themselves *pari passu* on the side originally healthy ; the symptoms were not therefore *relieved*, in a proper sense of the word, but merely *displaced*, the amount of disease in the body, if we can employ the figure, remaining the same. When, after a while, the symptoms had returned to their original site (*anæsthesie de retour* of Burq), they were observed for a time to *oscillate*—that is, diminishing degrees of anæsthesia continued rhythmically to present themselves on the originally sound side for some time, balanced by corresponding returns of sensation to the side originally anæsthetic, until at last matters stood exactly as before the operation. This whole description is vividly suggestive of the manner in which a temporarily displaced pendulum returns to equilibrium. Still further, Charcot described the process as follows : Given a case of left hemianæsthesia with a metal applied to the left forearm. At the moment when sensibility is appearing at the border of the metal, it is also found to be appearing in a somewhat analogous area on the left leg ; over corresponding areas on the right arm and leg (originally healthy) anæsthesia begins to show itself. These areas spread until at last sensation has been restored to the left side and the right side is completely anæsthetic ; this *transfer*, as has been said, includes also the other nervous disturbances associated with the hemianæsthesia—taste, smell, hearing, muscular power, temperature, etc. So much for *æsthesiogenesis*, *transfer*, *oscillations ;* another phenomenon is *produced anæsthesia* (anæsthesie provoquee). To illustrate it : Given a case of hemianæsthesia ; apply a metal, previously found suitable to the case, to the sound side. Immediately anæsthesia develops on the sound side to which the metal is applied, while sensation returns to the affected side. This is *produced anæsthesia ;* it has a prognostic value, as follows : Given a patient with hysterical hemianæsthesia, whose symptons have disappeared, and who is supposed to be cured. Apply a metal to which she used to be susceptible to the side originally sound. If any anæsthesia is produced, then the hysterical diathesis is not yet cured, and the patient is still liable to relapses. The discovery of *produced anæsthesia* renders possible the following generalization: 1. Æsthesiogenetic action is essentially bilateral. 2. It has for an immediate and invariable result the placing of sensibility (and other connected functions) in a state of activity the converse of that which was originally present. In hysterical cases æsthesiogenics are powerless when a paroxysm is impending. Another strange fact is the following: If, while a patient is experiencing the influence of a metal to which she is susceptible, another metal to which she is *not* susceptible be superimposed, matters are brought to a standstill and remain *in statu quo* almost indefinitely, or until the metal is removed. If a third metal, to which she *is* susceptible, be still superimposed, the patient responds to its influence as if the first and second were not present. Æsthesiogenics have been used in cases not hysterical; the cases are few, but the results seem to have been the same, with two exceptions—there is no *transfer*, and improvement persists to a greater or less degree.

The author proceeds to speak of different theories which have been advanced in the effort to explain the phenomena which have been described. The most prominent are: 1. Skepticism in various degrees, viz.: *a.* Entire disbelief of the facts noted, attributing them to errors of observation, and considering hysteria to be nothing more than a morbid disposition in young women to deceive themselves or others, or both. *b.* Another form of skepticism is that which explains all that has been described by the hypothesis of *expectant attention*, so-called. This idea, first advanced by John Hunter to explain mesmerism, consists mainly in the fact that if a person intensely fixes his attention upon a part, and imagines that he will feel in it warmth, cold, pain, etc., he soon does seem to feel the expected sensation, in a greater or less degree. Such phenomena would be accentuated in hysterical patients, and it is in these that the effects of metalloscopy are most distinct. The author, in this connection, remarks that he will not answer those prejudiced individuals who profess to doubt entirely; to those who ascribe everything to imagination, he answers that if this were the case it would be easy to prove it by changing the metals, the poles of the battery, etc., without the knowledge of the patients. This system of investigation has been thoroughly followed out in La Salpetriere, but only with the result of rendering the reality of the phenomena more apparent. 2. The hypothesis of an electric action in the case of metals at once suggests itself. To prove this, two plates of the same metal were applied, previously connected by a wire and galvanometer; in this way the fact was established that a feeble current was generated. And it was further proved that a galvanic current of the same strength reproduced the phenomena following the application of the metals. To extend these results to make them apply to the case of a single metal plate being used, it is necessary to assume that the lack of homogeneity in the plate is sufficient to admit of the generation of currents between different parts of the same plate. Unfortunately, these experiments were made before anything was known of *transfer* and *oscillations*; they, therefore, require repetition. To thus explain the metalloscopic phenomena, it is necessary to assume that the metal and body constitute a Voltaic couple, the body being qualified to act by virtue of being largely composed of solutions of chlorides, which are known to be active in this connection. 3. *Static electricity* is the best and most universally applicable æsthesiogenic. No special explanation of its mode of action in this connection is attempted. 4. The application of a *tuning-fork* in such a way that its vibrations are communicated to the part to be affected is among the most effective modes of æsthesiogenesis. This is not explained. 5. *Magnets* are very effective, and a feeble one is as efficient in this connection as the most powerful. Maggiorani says that the influence of a magnet may be exerted satisfactorily even when it is in the physician's pocket, his hand being upon it, while with the other he pretends to be counting his patient's pulse. Not explained. 6. Various chemical agents, such as solutions of mineral salts, sinapisms, vesicants, etc., have more or less æsthesiogenetic action. Their action is not explained.

The author says that the phenomena of æsthesiogenesis, as presenting themselves in hysterical patients, differ only in degree from those occurring in individuals with organic lesions and other nervous diseases than hysteria. He therefore considers that, while it is convenient to study the subject among hysterical patients, the results of his investigations admit of more or less general application. He believes that this is the first great step toward a system of *physical* medicine by which the physiological and pathological processes may be profoundly influenced and controlled in a way which it is difficult to conceive of in the present state of our knowledge.

